# Living with Parkinson's and the Emerging Role of Occupational Therapy

**DOI:** 10.1155/2015/196303

**Published:** 2015-10-01

**Authors:** Jelka Jansa, Ana Aragon

**Affiliations:** ^1^Neurologic Hospital, University Medical Centre Ljubljana, Zaloska 2, 1000 Ljubljana, Slovenia; ^2^Phoenix Cottage, New Buildings, Carlingcott, Bath, UK

## Abstract

Parkinson's disease is a chronic and increasingly complex condition, demanding multidisciplinary management. Over the last twenty years or so, alongside the growth of specialist services and healthcare teams specifically developed for people with Parkinson's, occupational therapy has grown in recognition as a treatment option, especially since evidence of its efficacy is now slowly emerging. The purpose of this work is to outline the role of occupational therapy clinical practice in the management of people living with Parkinson's disease and its emergent evidence base, combined with details of current occupational therapy philosophy and process, as applicable to occupational therapy practice for people with Parkinson's. The Canadian Practice Process Framework is used to structure this overview and was selected because it is a well-recognized, evidence-based tool used by occupational therapists and encompasses the core concepts of human occupation and person-centred practice. The framework employed allows the flexibility to reflect the pragmatic occupational therapy intervention process and so enables the illustration of the individually tailored approach required to accommodate to the complex pathology and personal, domestic, and social impacts, affecting the functioning of Parkinson's disease patients on a daily basis.

## 1. Introduction

Parkinson's disease (PD) develops in an insidious and increasingly complex manner and, until a cure is devised, must be endured by affected individuals for the remainder of their life spans. Although progressive by nature, Parkinson's is not of itself terminal; hence, over the often prolonged time span of the condition, numerous domains of every day function including motor and nonmotor performance are affected. In addition to numerous characteristic symptoms, the manifestation of the disease is influenced by environmental conditions, often fluctuates through the day, over the course of the disease, and is expressed in a highly individualised manner [1]. An occupational therapy process aims to address all of these issues, since the core of occupational therapy (OT) is* human occupation*, defined as “doing culturally meaningful work, play or daily living tasks, in the stream of time and, in the context of one's physical and social world” [2]. Systematic Cochrane reviews published in 2001 and 2007 [3, 4] concerning OT and Parkinson's found insufficient evidence to support or refute the value of OT for people with Parkinson's. However, national guidelines such as The National Parkinson's Disease Guidelines published in the UK in 2006 included a recommendation that OT is available* as required* to people with Parkinson's from the time of their diagnosis [5].

This overview explores the philosophy and evolution of the OT role specifically in the management of people with Parkinson's from the unique perspective of the two authors, who have a combined 47 years' professional experience of working with people living with Parkinson's (and related movement disorders). Recently published, robust and rigorous randomised controlled trials (RCTs), along with earlier non-peer reviewed, published sources that synthesised and translated basic research evidence into clinical practice, and more recent evidence-based Parkinson's specific guidance for OTs are also presented. This emergent evidence base underpins and demonstrates the role of OT and its value, in terms of health related quality of life benefits of OT for people with Parkinson's and in some cases for their care givers as well.

A patient-centred, top-down approach, which is initially focused on functional performance problems, is fundamental to OT assessment and intervention, as noted by Neistadt [6] as this approach helps to immediately identify functional performance problems of concern to patients and focus an OT's attention quickly on those impairments that are causing functional difficulties. Two well-established models employed to underpin OT practice support this mode of clinical reasoning and working practice, namely, the* Occupational Therapy Intervention Process Model* [7] and the* Canadian Practice Process Framework* (CPPF) [8]. These models can be applied to any medical diagnosis, including PD, and the CPPF has been selected to structure our content.

## 2. Canadian Practice Process Framework

The CPPF is evidence-based and encompasses the core concepts of* occupation* and* patient-centred practice*. Also incorporated are key concepts concerning* enablement* and an* occupation-based* focus, and additionally the CPPF takes into account the following contexts:* societal, clinical practice, the frame of reference*, and* the actual process*, as represented by eight action points which will be explored here and are listed below [8]:

1. Enter/Initiate.
2. Set the stage.
3. Assess/evaluate.
4. Agree objective and plans.
5. Implement plan.
6. Monitor/modify.
7. Evaluate outcome.
8. Conclude/exit.

### 2.1. The Frame of Reference, Societal, and Practice Context

The underlying clinical reasoning of an occupational therapist, dealing with PD patients, should be focused on facilitating performance of personally meaningful everyday occupations and addressing issues that interfere with their performance. Reasons for impaired or unsatisfactory performance can include stage of the disease, environmental restrictions, both physical and social, drug regimes, motivational issues, fatigue, depression, cognitive impairments, and motor symptoms such as freezing, plus other motor impairments. As every individual experiences Parkinson's in their own unique manner, it is not standard practice to limit OT interventions by focusing on prescribed goals and symptoms or according only to the stage of the disease. Disease specific staging models such as the 5-point Hoehn and Yahr scale (H&Y) [9, 10] offer a simple snapshot of functional impairment and disability [10]. In addition to awareness of the stage of the disease, OT intervention must also consider personal concerns, abilities, and goals. Therefore, OTs must utilise both conceptual models and models of practice simultaneously. Conceptual models support clinical reasoning about PD patients and their occupations and environments [11]. Meanwhile, to enable OTs to intervene tangibly, on a practical and pragmatic level, models of practice help OTs to promote beneficial changes in PD patients' daily activities, engagement in meaningful occupations, and to participate more fully in their physical environments. Conceptual models used by occupational therapists working with patients with PD include the Model of Human Occupation [2], the Person Environment Occupation (PEO) model [12], the Canadian Model of Occupational Performance-Enablement (CMOP-E) [13], and others. Frames of reference and practice models are connected, because frame of reference will change [8] with the demands of “Parkinson's patient-occupational therapist” interactions; thus frames of reference and practice models are combined to support the clinical practice process.

OT intervention strategies designed specifically for people with Parkinson's are often based on teaching the use of cognitive and sensory attentional strategies. Such coping strategies can be taught to interested patients to aid almost any daily task such as getting dressed more easily and undertaking safer and easier transfers and mobility around the home. This approach was first suggested in 1997 by a multidisciplinary team based in Melbourne, Australia, in their pioneering book:* Parkinson's Disease: A Team Approach* [14]. The role of OT in the management of people with Parkinson's disease was also reported in 1998 by an OT based in the UK [15]. Subsequently, a meta-analytic review published in 2001 by a team in the United States evaluated the effectiveness of occupational therapy-related treatments for people with Parkinson's disease and concluded that whilst further evidence was called for, the team reported that “to provide effective occupational therapy intervention, it is necessary to draw on research evidence” and suggested their own review as “a source of evidence for occupational therapists who treat clients with Parkinson's disease” [16].

Since publication in 2006 of the UK National Institute for Clinical Excellence (NICE) Clinical Guidelines for Parkinson's Disease, there has been a growing trend for using rehabilitation as an adjunct to pharmacological and surgical treatments, with an emphasis on the importance of multidisciplinary management of this highly complex and multidimensional condition. The 2006 NICE recommendation specifically addressing the role of OT for people with Parkinson's [5] highlights the need for consideration during OT assessment of work, family, leisure, transfers, mobility, personal self-care (such as eating and drinking, washing, and dressing), environmental safety, motor function, and cognitive function and recommended provision of appropriate OT interventions in these domains. NICE's 2006 recommendation for OT [5] was based on just 2 small scale and rather dissimilar group therapy RCTs [17, 18], yet the role of OT was given additional weight by the expert guideline development group [5]. However, NICE (2006) does not offer advice for OTs about* how* to work specifically with people who have Parkinson's, yet clinical experience shows that standard, generic OT approaches yield little benefit with this particular group of patients. The often paradoxical and counterintuitive responses of people with Parkinson's to generic OT interventions may be due to the complex neurological causes underlying the functional impairments experienced by these particular patients. In 2003 following a 2-wave national survey of around 160 OTs in the UK, Deane and colleagues had already suggested the need for postgraduate training for occupational therapists, who had acknowledged some of the unique challenges of treating Parkinson's patients a few years before publication of the 2006 NICE guidelines [19, 20]. To support OTs working with people with PD, Parkinson's UK (a national charity) and the College of Occupational Therapists collaborated to develop the* Occupational Therapy for People with Parkinson's Best Practice Guidelines* published in 2010 in the UK (and available since then online), based mainly on basic scientific findings, expert opinion, and clinical experience and subsequently peer reviewed and ratified by over 30 OTs [21].

Occupational therapists, working with people who have PD, should have access to knowledge and skills specifically concerning Parkinson's and be aware of resources and developments in this area of OT practice. The findings of an online survey published in 2011 about the views and experiences of OT of 230 people living with PD in 4 European countries (Norway, Slovenia, Sweden, and the UK) demonstrate that participants had numerous concerns regarding daily functioning and OT was considered as an important and highly valued intervention by the 54% of the survey participants who had received an OT service since their diagnosis of Parkinson's [22]. According to Sturkenboom et al. [23, 24], a recent high quality RCT and subsequent economic evaluation of OT in The Netherlands involving 191 community dwelling Parkinson's patients and 180 care givers led to measurable improvements in self-perceived performance in daily activities (as shown by improved scores on the COPM used as a primary, patient-centred outcome), without adding to overall healthcare costs. As a 2 to 1 randomisation method was employed, 124 participants received 10 weeks of individualised home-based OT intervention and 67 controls had only usual medical care. Sturkenboom et al. [23, 24] show that OT enabled “smart spending” of healthcare funds yielding personally significant gains in domains which have a direct influence on quality of life. Furthermore, OT led to positive cost-effective support for caregivers of people with PD [24]. There is also evidence published in 2010 from a groundbreaking RCT conducted in the United States suggesting that people with PD (*n* = 117) benefit in terms of their health related quality of life from self-management rehabilitation delivered in a group setting, at 3 intensities, and at 2 levels and according to disease stage, by a team of OT, physiotherapy, and speech therapists experienced in working with Parkinson's [25]. Most gains were demonstrated in terms of activities of daily living (ADL) when patients identified more concerns with ADL and mobility at the initial assessment stage [25]. The same research group published a systematic review in 2014 of interventions used by OTs with Parkinson's patients in which the 3 broad categories of OT intervention evaluated were (a) exercise or physical activity, (b) environmental cues, stimuli, and objects; and (c) self-management and cognitive-behavioural strategies. Moderate to strong evidence was found for task-specific, targeted physical activity training—on motor performance, postural stability, and balance. Evidence of moderate quality also demonstrated positive effects on motor control of external supports used during functional mobility or other movement activities. In addition, moderate evidence was found to support individualized interventions focused on promoting participant wellness and personal control by means of cognitive-behavioural strategies, to improve targeted domains of quality of life [26]. This adds support to Rao, also based in the United States, whose 2010 update of OT and Parkinson's [27] also demonstrated benefits for people with PD in terms of mobility and health related quality of life. Very recent evidence employing neurorobitcs and gaming technology (exergaming) for rehabilitation (with origins dating back to 2002), applicable to people with all neurological conditions, including PD, has been published [28–32] adding further depth and a new dimension to this evolving field of clinical practice. Further evidence about how OTs can use these novel approaches for the benefit of people with PD will no doubt emerge in the near future.

As a part of the OT assessment process, meaningful information about the patients' home and working environments as well as social and community support opportunities is gathered. PD patients' knowledge and experiences should be explicitly integrated into the process of evidence-based clinical decision-making in occupational therapy [16]. Personal strengths might include attributes, abilities, values, and beliefs, both spiritual and cultural [11]. Strengths and challenges may also be identified by the involvement and support of “significant others” in the patients' life, especially during H&Y stages 2–5 of the disease. Within this initial stage of the OT process, the OT must also search for, identify, and provide reliable information that empowers patients and enables their families to become knowledgeable about their condition before engaging in therapeutic interventions. The methods and key elements of this OT process will now be detailed in the following 8-point action plan.

### 2.2. Eight Action Points

#### 2.2.1. Enter/Initiate

Based on a healthcare or social care referral or by private contract, an OT process is initiated and an occupational therapist would initially consider their own professional competencies and experiences to work with patients with PD and if indicated may decide not to continue the OT process [8]. Generally though an OT will proceed to identify potential/actual occupational issues. Evidence available to date suggests that OT intervention is most often required in the intermediate to advanced stages of Parkinson's equivalent to H&Y stages 3–5 [33–35], whereas it is likely to be of benefit to introduce OT earlier (H&Y stages 1 and 2) as a form of secondary prevention [22, 23, 36]. Standard healthcare practice lags behind this ideal, however, as seen, for example, from a Dutch survey of healthcare for people with Parkinson's, which revealed that only 9% of the PD population received OT consultation [37].

#### 2.2.2. Set the Stage

The person-centred nature of OT acknowledges the individual as the central element of the treatment process [38]. Thus, OT intervention will be most effective when methods to identify those occupational issues that are most important to PD patients are employed. The Canadian Occupational Performance Measure (COPM) [39] was designed to identify an individual's perceptions of his or her performance in daily activities over time. The COPM is a standardized, semistructured interview tool that is cost-effective, easy to administer, and sensitive to change [1]. It has been used widely in OT research, and in a recent study by Sturkenboom et al. [23] where COPM was used as a primary outcome measure it showed good sensitivity and ability to capture the efficacy of community OT for people with PD. While conducting a COPM interview, an OT will ask a patient with PD to identify the activities that are difficult to perform in the domains of self-care, productivity, and leisure [6]. Further to this, a person with PD will be asked to identify up to five of their highest priority problems and to rate his or her performance and level of satisfaction in each of these activities. The occupational therapist listens to the PD patient's story within the context of their personal, physical, and social environment. Use of COPM assists a person with PD to identify those daily tasks that he/she wants to do, needs to do, or is expected to do, but can not or that are not performed to a satisfactory level for the individual [11]. This approach presents the unique concerns of OT [40], thus ensuring efficiency and promoting increased effectiveness of OT intervention with PD patients, and also captures the unique domain/focus of occupational therapy [7]. Other examples of approaches to engage a PD patient in the OT process could be by standard interview using structured, semistructured, or open interview methods, or by using Goal Attainment Scaling.

#### 2.2.3. Assess/Evaluate

Some assessment scales utilised by OTs in their clinical practice with Parkinson's patients are disease specific and have been devised only for use in PD; other scales such as COPM are applicable to a wide range of medical conditions and have been validated for PD. During the OT assessment process, it is important to evaluate those impairments (motor and nonmotor symptoms) that have an impact on the performance of meaningful everyday tasks that the patient has identified, for example, by using the COPM. Impaired attributes may include challenges related to changes in manual dexterity, coordination and handwriting, visuospatial perception, cognitive skills, stamina (fatigue), mood (depression), motivation, pain, motor symptoms, and/or motor complications. According to concerns identified, there are several assessment scales that can be used to explore these domains further, including the Jebsen Test of Hand Function [41], modified by Jones and Harrison [42], which is a reliable and valid test of hand function. According to Jones and Harrison [42], Jebsen's Test of Hand Function can detect neurological deficit and measure changes in the severity of this deficit. Other useful assessments that may be used by OTs in their assessment process include the Parkinson fatigue scale [43], the Beck Depression Questionnaire [44], the Rivermead Behavioural Memory Test [45], and the Visual Analogue scale for the measurement of pain severity. There are several other assessment tools that can be used through this process. For example, when assessing ADL, The New Unified Parkinson's Disease Rating Scale ADL section can be employed [46] although it is important to note that the ADL section of this scale contains the mixture of impairment and disability-related items [47]. The Assessment of Motor and Process Skills (AMPS) is a performance-based, valid, and reliable measure of ADL, with no ceiling and floor effects [7]. AMPS can only be administered if the PD patient is able to engage in activities, and it is therefore not valid for those with extreme motor fluctuations or those in the later stage of Parkinson's disease [1]. For assessing driving ability in people with Parkinson's disease, Webster's Rating Scale differentiated between safe and unsafe drivers [48]; road testing however is probably the most sensitive assessment method, although driving ability also correlates well with ability on simulator testing. EuroQol is a valid and reliable measure of quality of life; in addition the PDQ 39 is another reliable and valid tool for measuring health related quality of life for those with Parkinson's disease [49].

Latest advances in robot-based and sensor-based measures are becoming increasingly available for OTs assessing temporal and spatial domains of bodily movement. For example, Parkinson's KinetiGraph (PKG) automatically records movement and can be worn continuously over 6 to 10 days during activities of daily living, so that, by using an accelerometer, movement data can be gathered in an automated manner to assesses bradykinesia and dyskinesia [50]. Another example of this leading edge approach is reported by Williams et al. [32] who explored freezing of upper limbs using sensors.

#### 2.2.4. Agree Objectives and Plan

Occupational therapist and PD patient's expectations should be focused towards the same achievable functional goals. It is important to negotiate goals that are specific and measurable and that can be achievable in the time frame available for intervention.

#### 2.2.5. Implement Plan

Intervention by an OT is individually tailored to each patient, resulting in an intervention plan that has personal relevance and meaning for the person and their family, and is therefore patient-centred [13]. OTs aim to maintain independence, confidence, and safety, in the performance of daily tasks and activities in all areas of life, for as long as possible. PD patients can benefit from OT from the time of diagnosis if daily tasks are problematic as shown by recent evidence [23, 34] that supports the value of OT in early stages of the disease. The role for OT as Parkinson's progresses increases. For example, as scores on the H&Y scale increase, especially when wearing-off problems, on-off fluctuations, dyskinesias, falls, and freezing are becoming more evident, it is important for OTs to educate Parkinson's patients about how to adapt their everyday routines and personal lifestyle, in order to optimise functional ability and health related quality of life.

The following types of treatment intervention [7] related to the H&Y stages could be used in the long-term management of Parkinson's disease (Table 1).

*Acquisitional occupation* is targeted towards the restoration of impaired skills [7] and is most relevant for the early stages of PD (relevant to H&Y stages 1–3). It relates to training/maintaining Parkinson's patients' quality of daily occupations.

*Restorative occupation* is targeted toward restoration of impaired personal factors (daily routines, values, and habits) and impaired bodily functions (motor, fatigue, balance, and motivation) [7]. Robot-assisted arm training is less studied in Parkinson's, yet it offers another promising tool for improving upper limb function in this condition [29]. Furthermore, the use of exergaming technology such as Nintendo Wii and Xbox Kinect are novel intervention tools that are increasingly being employed by OTs and other therapists within neurorehabilitation services [28, 30, 31]. These novel exergaming tools are used to facilitate different types of exercise, with the aim of improving balance and reaction times. Exergaming devices have the added advantage that they can be used both in institutions and in the home setting [30, 31].

*Adaptive occupation* [7] is targeted toward adapted methods of performing daily occupations; interventions also include the introduction of aids and equipment and environmental adaptations. After analysis of PD patients' functioning and of their physical environment, the OT suggests methods and or equipment for safe, effective, and confident performance. This process could include teaching methods that help PD patients to perform daily occupations independently and in the most satisfying way that their residual ability allows, or the provision of hand rails, adapting bathroom facilities, installation of a stair lift, adapting the kitchen, supply of handwriting devices, adaptation of clothing, and so forth. Adaptive occupation is relevant to H&Y stages 2–5. As even in the advanced (H&Y = 5) stage, OT interventions can be devised to improve comfort, dignity, and enjoyment of leisure time.

Occupation-based educational programs delivered by OTs [7] involve implementation of workshops, lectures, or other educational programs for Parkinson's patients and/or their close relatives or care givers, related to the nature of Parkinson's, occupational issues, and other psychological and social concerns. These may include organisation or contributions by OTs to programs for newly diagnosed PD patients, patients at H&Y stages 2 and 3, and even patients in H&Y stages 4 and 5. Additionally, separate educational sessions or programs for relatives or care givers are available in some places.

Acquisitional, restorative, and adaptive occupation may be based on approaches that employ attentional strategies, cognitive and sensory cueing techniques, and conductive education methods, which will be outlined below.

*Conductive education* originally developed for use with children affected by cerebral palsy usually employs group-based exercises using rhythmically facilitated practice of basic movements, which are required for daily living tasks, and is applicable to PD patients [51]. This approach is used, for example, to improve handwriting, gait, and other forms of movement control.

*Cueing techniques* using a personalised selection from a wide variety of cognitive and sensory stimuli can aid preparation for and performance of personal, domestic, and community activities by people living with PD [52]. In an international collaboration with many leading authorities in the field, a book entitled* Rehabilitation for Movement Disorders* (including Parkinson's disease) was published in 2013 and edited by Iansek and Morris. This work translated basic laboratory-based research from a wide array of high-tech, brain imaging studies (e.g., fMRI, SPECT, and PET) [52]. Iansek and Morris suggest in the introductory section of their book that the use of attention, combined with specific cognitive processes and sensory cues, allows a person with PD (or their care giver) to exploit alternative short motor loops (that can be engaged for survival) and thus may enable access to available neural pathways, structures, and neurotransmitters to enable improved performance [52]. Cues appear to “by-pass” the underactive basal ganglia (or autopilot system) and to thus elicit and enhance the performance of functional activities by employing alternative “manual control” mechanisms [52]. In an interesting RCT also from Morris's Australian team, external cues and attentional strategies aimed to improve size, speed, and movement sequence were also found to have a positive effect on ADL performance [53]. Applying and building on these findings in relation to handwriting, which is often a great frustration for people with Parkinson's, OTs can also be informed by a well conducted yet small RCT of Micrographia by Oliveira et al. [54] from the UK who showed back in their 1997 publication, that both external visual and auditory cues draw attention to the goal of writing bigger and thus encourage the patient to write less automatically, with the beneficial effect of increasing amplitude of handwriting. Two further handwriting studies published in 2005 and 2007 [55, 56] also showed that motor performance can be aided by the cognitive/sensory strategy of prior prereading of a word, or by seeing a word semantically related to the expected motor performance. Thus seeing the word “Reach” on a prompt card, for example, or saying the word “Reach” stimulates the neural networks responsible for the performance of a reaching task.

Motivational Interviewing may also be practised within an OT process to promote adherence to a treatment plan. A review by O'Halloran et al. [57] looked at the use of Motivational Interviewing amongst people with chronic health conditions and demonstrated that approximately 75% of the studies examined demonstrated a beneficial effect, regardless of whether the problems being addressed were psychological or physiological. The same authors argue that Motivational Interviewing is not limited in any way to counselling of a small group of selected clients but can be used in the treatment of a broader area of diseases that, to some extent, are influenced by behaviour, and go on to suggest that this is a method with an important potential effect, from which patients may very well benefit [57].

#### 2.2.6. Monitor/Modify

It is important to monitor and modify the OT intervention process at frequent points during a course of OT sessions. To fully engage each PD patient, it is beneficial to follow their personal agenda of key concerns, and working collaboratively with the patient is critical to the success of the OT intervention process. As applicable, an OT intervention plan is reviewed, redesigned, and adjusted according to progress in addressing personally relevant goals. Often, as issues are resolved or an acceptable management approach has been established, new priorities arise and the OT must therefore anticipate and monitor new concerns and goals through ongoing evaluation.

#### 2.2.7. Evaluate Outcome

Evaluation can identify whether the OT process has facilitated attainment of goals, development of life skills, and adaptation to the progression of the disease. Thus, evaluation assists the process of healthy occupational adaptation for the PD patient. According to the assessments, evaluation, and goal setting methods used to establish the OT process, repeated measures can be undertaken as a course of OT progresses and in particular, as an episode of care nears conclusion.

#### 2.2.8. Conclude/Exit

The therapist and the PD patient will need to communicate about the conclusion of the OT process as an episode of care ends. Because self-management is promoted throughout the rehabilitative process, dependence on the OT is not encouraged or is contained. Often people with Parkinson's will return for further goal-focused courses of OT intervention as their symptoms change, the condition progresses, or other comorbidities or life events necessitate another course of OT intervention. Following each episode of OT care, a written or oral report is provided for patients and is shared if appropriate with other multidisciplinary team members. People with Parkinson's and their families or care givers should be provided with adequate information for transfer to other supportive services and for reentry to OT as required.

## 3. Summary

The CPPF has been used here to demonstrate how Parkinson's patients who engage in an OT intervention process are enabled through a flexible, individually tailored approach to accommodate to the complex pathology and functional impacts they face in living with PD. As an OT intervention proceeds, it must be adjusted to each Parkinson's patient's current needs, and thus it is useful to use relevant contexts and stages from the eight action points outlined above. In concordance with Davis et al. [8], this way of reasoning about the OT process has been employed to make it possible for some less observable aspects of OT practice to be made explicit. The growing evidence base presented here also shows that referral for OT assessment and intervention is relevant for Parkinson's patients willing to engage in a suitably tailored course of OT intervention, at any stage of the condition (H&Y stages 1–5).

## 4. Conclusion

The role of OT for people with Parkinson's, ideally delivered within the context of a multidisciplinary healthcare team, is now becoming well established. High quality research to build on the firm foundations explored here would be of particular interest in establishing more details about the effects of OT intervention. Studies of OT (and other rehabilitation therapies) used to address functional concerns about activities of daily living before commencement of anti-Parkinson's medications would be of particular interest. Establishing the value of OT, using a Parkinson's specific approach as outlined here and specific studies focused on the short-, medium-, and long-term value of OT, for example, commenced in the early (H&Y stages 1-2) stages of Parkinson's, would also add to the small but rapidly growing evidence base concerning rehabilitation of people with PD. This information may be of particular use to OTs facing the many challenges of their Parkinson's patients. Furthermore those diagnosed with Parkinson's at a younger age and others who wish to find nonpharmacological ways to delay or minimise reliance on anti-Parkinson's medications may also benefit by increased understanding of the value of participation in intermittent episodes of OT intervention.

## Figures and Tables

**Table 1:** OT treatment intervention modes as related to the Hoehn and Yahr scale [9].

Hoehn and Yahr stage	OT treatment intervention modes [7]

Stage 1	Acquisitional occupation, restorative occupation, and occupation-based education programs

Stage 2	Acquisitional occupation, restorative occupation, occupation-based education programs, and adaptive occupation

Stage 3	Acquisitional occupation, restorative occupation, occupation-based education programs, and adaptive occupation

Stage 4	Adaptive occupation, restorative occupation, acquisitional occupation, and occupation-based education programs

Stage 5	Adaptive occupation, occupation-based education programs
